# Application of large-scale targeted sequencing to distinguish multiple lung primary tumors from intrapulmonary metastases

**DOI:** 10.1038/s41598-020-75935-4

**Published:** 2020-11-02

**Authors:** Jiaxin Duan, Mingjian Ge, Jian Peng, Yangli Zhang, Li Yang, Ting Wang, Tian Qin, Rui Yuan, Yuhong Zhang, Wei Cheng

**Affiliations:** 1 The Center for Clinical Molecular Medical Detection, The First Affiliated Hospital of Chongqing Medical University, Chongqing, 400016 People’s Republic of China; 2 Department of Cardiothoracic Surgery, The First Affiliated Hospital of Chongqing Medical University, Chongqing, 400016 People’s Republic of China; 3 Department of Respiratory and Critical Care Medicine, The First Affiliated Hospital of Chongqing Medical University, Chongqing, 400016 People’s Republic of China; 4 Burning Rock Biotech, Guangzhou, 510300 People’s Republic of China; 5 Key Laboratory of Biorheological Science and Technology of Ministry of Education, School of Bioengineering, Chongqing University, Chongqing, 400044 People’s Republic of China

**Keywords:** Cancer, Computational biology and bioinformatics, Molecular biology, Medical research, Molecular medicine

## Abstract

The effective differentiation between multiple primary lung tumors (MPs) and intrapulmonary metastases (IMs) in patients is imperative to discover the exact disease stage and to select the most appropriate treatment. In this study, the authors was to evaluate the efficacy and validity of large-scale targeted sequencing (LSTS) as a supplement to estimate whether multifocal lung cancers (MLCs) are primary or metastatic. Targeted sequencing of 520 cancer-related oncogenes was performed on 36 distinct tumors from 16 patients with MPs. Pairing analysis was performed to evaluate the somatic mutation pattern of MLCs in each patient. A total of 25 tumor pairs from 16 patients were sequenced, 88% (n = 22) of which were classified as MPs by LSTS, consistent with clinical diagnosis. One tumor pair from a patient with lymph node metastases had highly consistent somatic mutation profiles, thus predicted as a primary-metastatic pair. In addition, some matched mutations were observed in the remaining two paired ground-glass nodules (GGNs) and classified as high-probability IMs by LSTS. Our study revealed that LSTS can potentially facilitate the distinction of MPs from IMs. In addition, our results provide new genomic evidence of the presence of cancer invasion in GGNs, even pure GGNs.

## Introduction

According to GLOBOCAN’s 2018 database statistics, lung cancer morbidity and mortality is 11.6% and 18.4%, respectively, remaining at the top of the cancer list^1,2^. Recently, with the development of radiological technology, an increasing number of lung cancer patients are diagnosed as carrying two (or more) malignant lesions, called multifocal lung cancers (MLCs), characterized by ambiguous staging. It is generally known that the treatment of multiple primary lung cancers is the same as the one used against single primary lung cancers. However, the identification of multiple primary lung tumors (MPs) and intrapulmonary metastases (IMs) is crucial in clinic practice, because IM is the malignant lung tumor in its advanced stage and a palliative treatment is generally adopted^3^. At present, most of the clinical decisions are based on the comprehensive histologic assessment^4^, which is empirically derived and affected by interobserver variability. As long as the types of tissue are different, the MLCs can be diagnosed as primary cancers. Nevertheless, if the histological types and subtypes are the same, more clinical information is needed to coordinate^5^. Although the diagnostic criteria to recognize MLCs have been proposed and improved over the years, reliable and effective methods are still lacking to clearly distinguish IMs from MPs^6^.

From the perspective of evolutionary biology, the development of tumors follows Darwin’s theory of natural selection, choosing a suitable way in both expansions and constraints^7–10^. The continual appearance of random mutagenesis and universal selection of positive and negative induce the distinct tumorigenic alterations of each lesion^7,11^. Sequencing technologies can effectively identify the clonality of multiple lesions in the same patient affected by certain genetic changes to distinguish the primary lesions from the metastatic ones^12–16^. As reported in previous studies, the molecular spectrum of independent clones is quite different, and the lesions with great similarity in the mutation spectrum tend to be IMs^7,17,18^. To date, somatic mutations in several major driver genes^12^, targeted sequencing for dozens of genes^13–15^, loss of heterozygosity^19^, and chromosomal rearrangements^20^ have been applied to discriminate MPs from IMs. Although these manners provide valuable information about tumor clonal relationships, the number of genes in the test method may affect the differential diagnosis of MLCs. A larger gene panel could enhance the efficiency of lineage calling and the sensitivity of differentiated diagnosis^20,21^. Most recently, whole-exome sequencing (WES) and whole-genome sequencing (WGS) have also been proposed to delineate the clonal relationships among different tumors^7,16^. There is no doubt that WES and WGS can provide pivotal information to accurately distinguish MPs from IMs, but it is unrealistic to apply them to every single tumor in clinical practice^22,23^. Hence, the clinical value of large-scale targeted sequencing (LSTS) should be further explored and confirmed.

Therefore, in this study, a LSTS assay covering up to 520 cancer-related genes was performed on 36 tumor samples from 16 patients diagnosed with multiple primary lung cancers. Subsequently, mutational profiles were combined with clinical, radiological, and histopathological analysis to classify the paired tumors as MPs or IMs.

## Results

In this study, 36 lesions from 16 patients were investigated, which were diagnosed as MPs, including three patients who had three or more lesions (up to four). The characteristics of patients and tumors are summarized in Table S1. These tumors were differentiated into sarcomatous carcinoma (SC, 2.8%) and adenocarcinomas (ADC, 97.2%), and the latter including adenocarcinoma in situ (AIS, 2.8%), minimal invasive adenocarcinoma (MIA, 11.1%) and invasive adenocarcinoma (IA, 83.3%) (Table 1).

**Table 1:** Clinical, CT and pathological data of patients with multiple lung cancers.

Patient-ID	Gender	Smoking status	Location	CT	Histology	Node staging
P1	Male	Former smoker	RUL	n/a	IA	IA3/T1cN0M0
			LUL	GGN	IA	IA2/T1bN0M0
P2	Male	Former smoker	RUL	n/a	IA	IIIA/T1cN2bM0
			LUL	n/a	SC	IIB/T3N0M0
P3	Male	Former smoker	RLL	Part-solid	IA	IB/T2aN0M0
			RUL	Part-solid	IA	IB/T2aN0M0
P4	Male	Former smoker	RUL	GGN	IA	IA3/T1c(2)N0M0
			RML	Solid	IA	IA3/T1c(2)N0M0
P5	Female	Non-smoker	LUL-I	Solid	IA	IIIA/T1bN2bM0
			LUL-U	GGN	IA	IIIA/T2aN2bM0
P6	Male	Non-smoker	RML	GGN	MIA	IA/T1aN0M0
			RUL	Part-solid	IA	IA/T1bN0M0
P7	Female	Non-smoker	LLL	GGN	AIS	IA/T1aN0M0
			LUL	Solid	IA	IA/T1aN0M0
P8	Female	Non-smoker	RUL	Solid	IA	IIA/T2aN1M0
			RLL	GGN	IA	IA/T1aN0M0
P9	Male	Non-smoker	LLL	Part-solid	IA	IA1/T1aN0M0
			RLL	Solid	IA	IA3/T1cN0M0
P10	Male	Former smoker	RLL-D	GGN	IA	n/a
			RML	Part-solid	IA	IB/T2aN0M0
			RUL-A	GGN	IA	n/a
			RUL	GGN	IA	n/a
P11	Female	Non-smoker	LUL	GGN	MIA	IA1/T1aN0M0
			RML	GGN	MIA	IA1/T1a(mi)N0M0
P12	Female	Non-smoker	RUL	GGN	IA	IA3 T1cN0M0
			LUL	GGN	IA	IA2 T1bN0M0
P13	Female	Non-smoker	RML	Part-solid	IA	IB/T2aN0M0
			RLL	n/a	IA	IA/T1aN0M0
			RLL-D	Part-solid	IA	IB/T2aN0M0
P14	Female	Non-smoker	RLL	Solid	IA	IA/T1aN0M0
			RUL	GGN	IA	IA/T1aN0M0
P15	Female	Non-smoker	RML	Solid	IA	IA1/T1aN0M0
			RUL	GGN	MIA	IB/T2aN0M0
P16	Female	Non-smoker	LUL	GGN	IA	IA1/T1aN0M0
			RML	GGN	IA	IA1/T1aN0M0
			RUL	GGN	IA	IA2/T1bN0M0

*IA* invasive adenocarcinoma, *AIS* adenocarcinoma in situ, *MIA* minimal invasive adenocarcinoma, *SC* sarcomatous carcinoma, *GGN* ground-glass nodule, *CT* computed tomography, *LLL* left lower lobe, *LUL* left upper lobe, *RLL* right lower lobe, *RML* right middle lobe, *RUL* right upper lobe, *LUL-U* the upper lingual segment of LUL, *LUL-I* the inferior lingual segment of LUL, *RLL-D* the dorsal segment of RLL; RUL-A, the anterior segment of RUL, *n/a* not applicable.

### Targeted sequencing statistics

**Figure 1:**
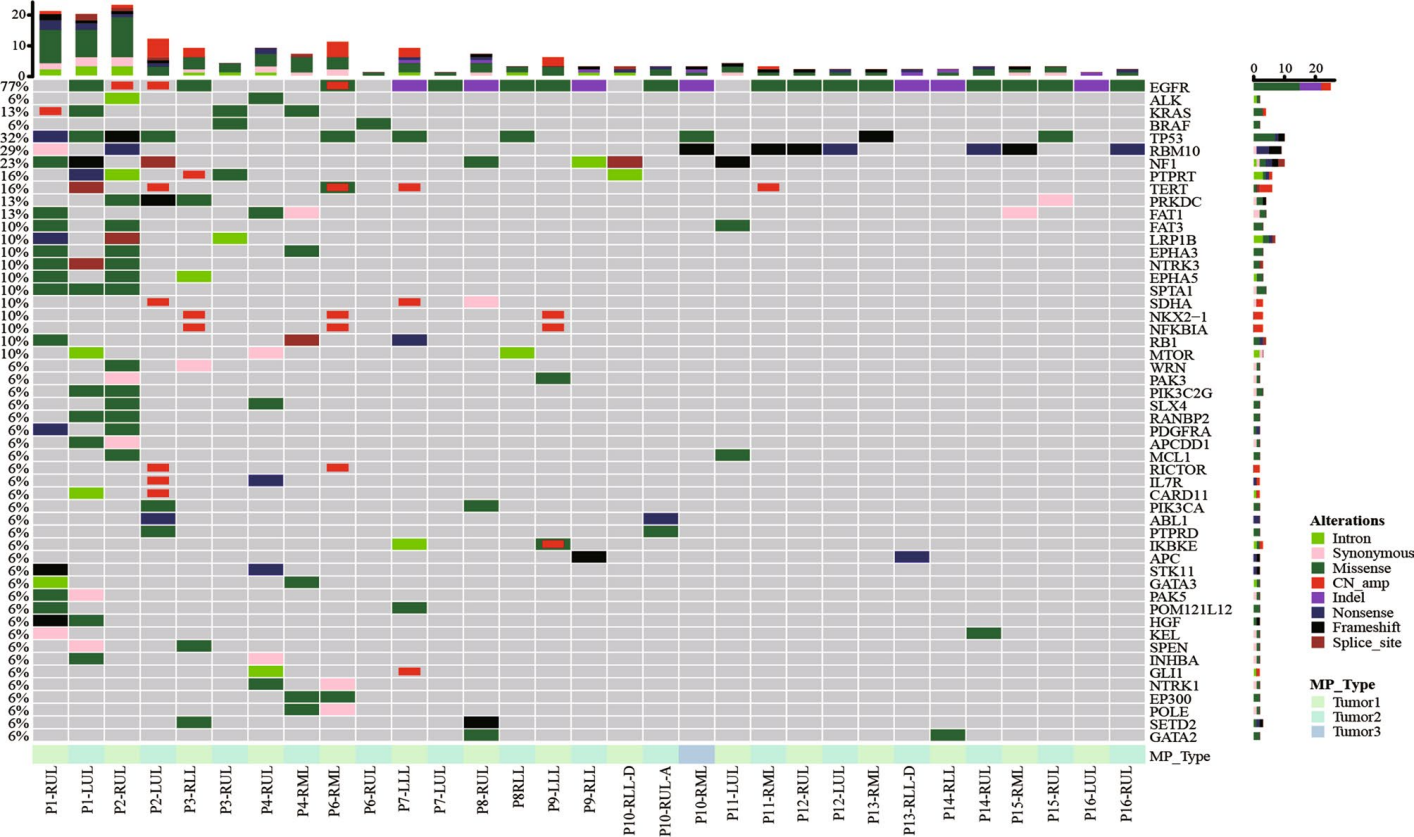
Oncoprint heatmap of variations in 35 tumors from 16 patients depicting the presence (see color legend) or absence (gray box) of specific mutation. Only genes with a detection rate ≥ 2 were included. *CN_amp* copy number amplification, *LGR* large genomic rearrangement.

**Figure 2:**
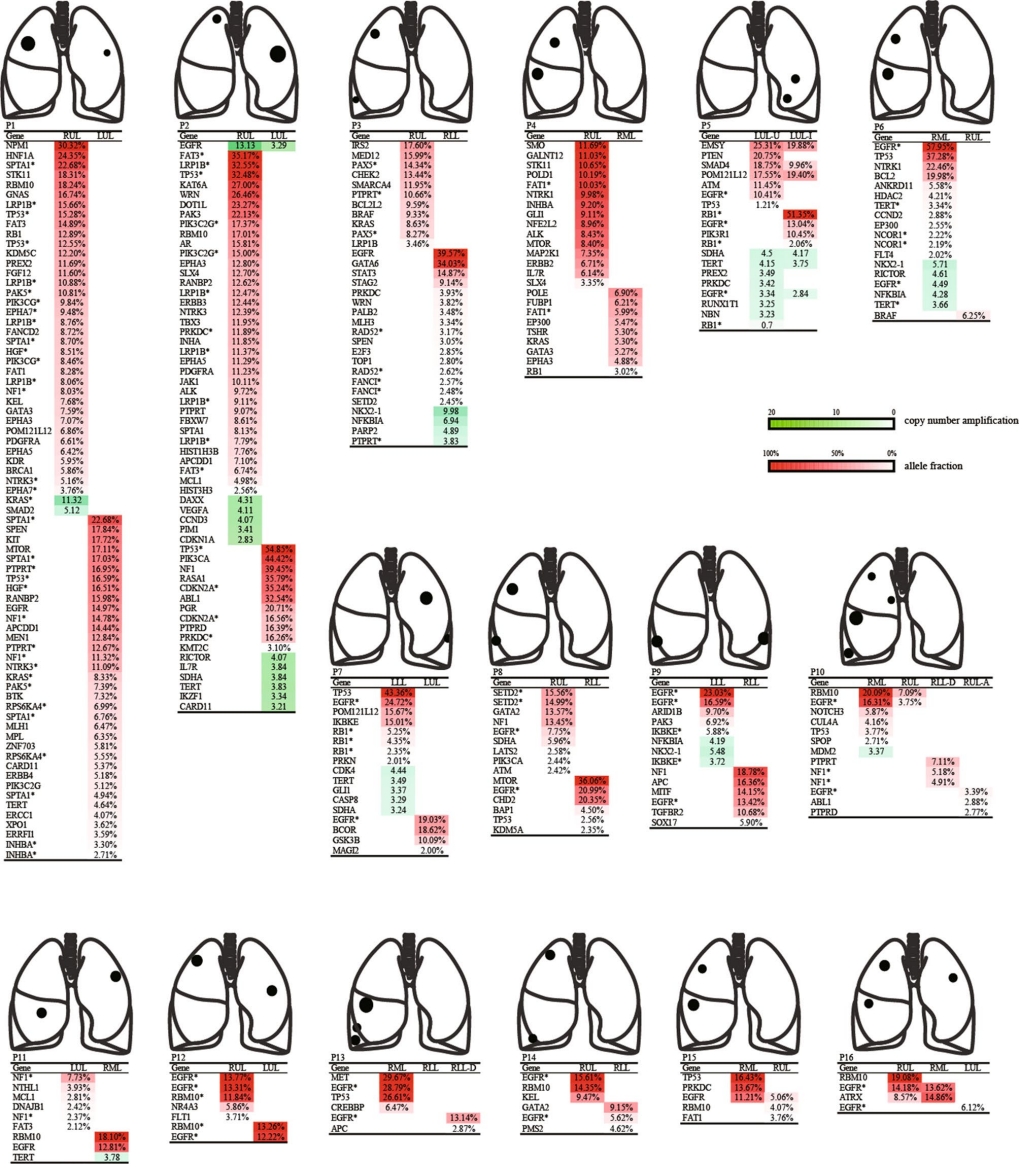
Heatmap of the gene variations in 16 patients with multiple lung cancers. This map shows the mutation spectrum of each tumor. Among these tumors, 33 lung cancers possessed distinct genomic profiles and three primary-metastatic pairs from three patients shared mutations (P5, P10, P16). Allele fraction and copy number amplification are depicted in red and green, respectively. *LLL* left lower lobe, *LUL* left upper lobe, *RLL* right lower lobe, *RML* right middle lobe, *RUL* right upper lobe, *LUL-U* the upper lingual segment of LUL, *LUL-I* the inferior lingual segment of LUL, *RLL-D* dorsal segment of RLL, *RUL-A* anterior segment of RUL. Asterisk (*) indicates different mutations in the same gene.

In order to validate the clinical application of LSTS to distinguish MPs from IMs, the comprehensive mutational profiles of 25 tumor pairs were analyzed using a panel of 520 cancer-related genes. Collectively, our analysis revealed 331 gene variations (286 mutations and 45 CNV) from 36 tumor samples, with a median of 9 somatic alterations per tumor (range 0–41). No gene variation was detected in one lesion of P13. A list of all variations detected by targeted sequencing is depicted in Table S3, from which Oncoprint heatmap (Fig. 1) and molecular profiling (Fig. 2) of the tumors from all patients were generated. *EGFR* alteration was the most frequently detected (77%), which was observed in 93.75% of patients (15/16), followed by *TP53* alteration (32%), consistent with a previous study on multiple lung cancers reported in 2018^24^. Notably, *RBM10* exhibited a significantly high mutation frequency (eight tumors, 25%) in stage I–II adenocarcinomas (32 tumors) (Fig. 1). The result was similar to the previous reports in which *RBM10* was found with a high mutation rate (16%) in radiological subsolid nodules^25^, and a high mutation rate (21%) in preinvasive and early-stage lung adenocarcinomas^26^. In addition, several oncogenes (such as *EGFR*, *SPTA*, and *RB1*) had different mutations in the same or different tumors on the same patient (Fig. 2). This phenomenon indicated both inter-tumor and intra-tumor heterogeneity of oncogenes in lung cancer.

### Mutational evaluation and tumor classification

No shared gene variations were detected between different tumors of 20 tumor pairs (75%), and these pairs were thus classified as definite MPs (Fig. 1, Table S4). In addition, *EGFR* was the only gene variation shared by two cases (CNV in P2, p.L858R in P15). And each of them also harbored some unique mutations, ranging from 2 to 40 mutations per tumor (Fig. 2). Given the prevalence of *EGFR*, the tumors in these two pairs were more likely independently arisen, leading to a clear classification of MPs with coincidental *EGFR* hotspot variation^27^.

Conversely, one tumor pair shared missense mutations in three genes (*EMSY*, *SMAD4*, and *POM121L12*) and gene copy number amplification of three genes (*SDHA*, *TERT*, and *EGFR*) (Fig. 2). These shared gene variations occurred in genes that were rarely reported, thus, this pair was classified as definite IMs.

Lastly, two tumor pairs shared two mutations. They were one pair (RML, RUL) in P10 shared mutations in *EGFR* (p.L747_T751del) and *RBM10* (p.V467fs), and one pair (RML, RUL) in P16 shared mutations in *EGFR* (p.L858R) and *ATRX* (p.S1012A) (Fig. 2). Except for p.L858R (22.8%), the deletion in exon 19 (including p.L747_T751del) is also a known hotspot mutation detected in *EGFR*, which accountes for 22.1% alone and 24.3% in combination with others^27^. Nevertheless, in The Cancer Genome Atlas (TCGA) database, approximately 6.5% of *ATRX* and 8.3% of *RBM10* mutations were reported in lung adenocarcinoma^28^. Based on the low prevalence of these genes, the odds of coincidental co-occurrence of {*EGFR* (p.L858R) and *ATRX* (p.S1012A)} or {*EGFR* (p.L747_T751del) and *RBM10* (p.V467fs)} in two independent tumors were lower than 2.20E − 04 and 3.36E − 04, repectively. Therefore, these two pairs were classified as high-probability IMs^29^.

### Comparison of clinical diagnosis to LSTS classification

Overall, 22/25 (88%) of the 25 tumor pairs matched the clinical diagnosis of MPs. However, three tumor pairs were discordant with the clinical diagnosis, including two high-probability IMs (shared two mutations), and one definite IM (shared six alterations).

**Figure 3:**
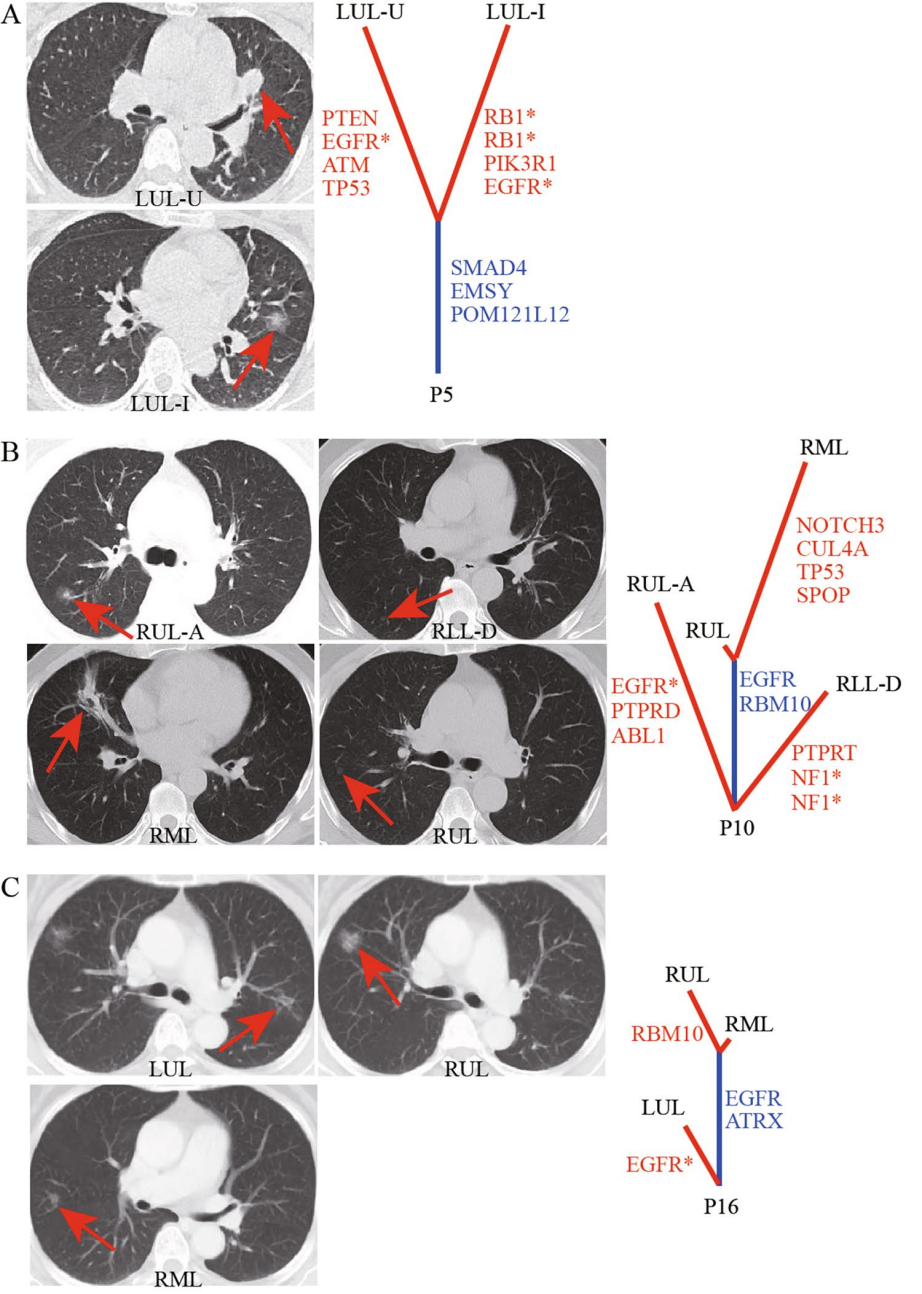
CT images and mutation spectra of patient 5, 10, and 16. Trunks (shared mutations) and branches (private mutations) were depicted in blue and red, respectively. (**A**) CT scans obtained with 0.6 mm-thick sections through LUL showed a solid nodule in the upper lingual segment and a GGN in the inferior lingual segment of patient 5. (**B**) CT scans obtained with 5 mm-thick sections through RUL-A, RLL-D, RML, RUL of patient 10 showed that all of them were ground-glass opacities, except the one in the RML, which was primarily solid consolidation accompanied with partially ground-glass opacity. (**C**) CT scans obtained with 5 mm-thick sections through LUL, RUL, RML of patient 16 showed that all of them were ground-glass opacities. *LUL* left upper lobe, *RUL-A* anterior segment of RUL, *RLL-D* dorsal segment of RLL, *RML* right middle lobe, *RUL* right upper lobe.

Tumors in the definite IM case (P5) were identified as adenocarcinoma by postoperative pathological examination. The proportion of histological subtypes showed that the acinar pattern (90%) was a significant subtype in one lesion, followed by papillary (5%) and micro-papillary (5%), while the subtypes of the other were papillary (75%), acinar (10%), adherent (10%), and micro-papillary patterns (5%) (Table 2). In addition, a 0.6 mm-thick CT slice revealed the presence of two nodules, one was pure GGN (pGGN), the other was a solid nodule (Fig. 3A). With the combination of pathological types with imaging data, these two lesions were diagnosed as separate primaries despite the presence of lymph node metastases.

**Table 2:** Histological subtypes and their percentage in the nine pulmonary lesions of patients 5, 10, and 16.

Patient-ID	Gender	Smoking status	Location	CT	Histology	Histological subtype				Node staging
						Acinar (%)	Papillary (%)	Micro-papillary (%)	Adherent (%)	
P5	Female	Non-smoker	LUL-I	GGN	IA	10	75	5	10	IIIA/T1bN2bM0
			LUL-U	Solid	IA	90	5	5	0	IIIA/T2aN2bM0
P10	Male	Former smoker	RLL-D	GGN	IA	100	0	0	0	n/a
			RML	Part-solid	IA	100	0	0	0	IB/T2aN0M0
			RUL-A	GGN	IA	100	0	0	0	n/a
			RUL	GGN	IA	100	0	0	0	n/a
P16	Female	Non-smoker	LUL	GGN	IA	100	0	0	0	IA1/T1aN0M0
			RML	GGN	IA	100	0	0	0	IA1/T1aN0M0
			RUL	GGN	IA	100	0	0	0	IA2/T1bN0M0

*IA* invasive adenocarcinoma, *CT* computed tomography, *GGN* ground-glass nodule, *LUL* left upper lobe, *RLL* right lower lobe, *RML* right middle lobe, *RUL* right upper lobe, *LUL-U* the upper lingual segment of LUL, *LUL-I* the inferior lingual segment of LUL, *RLL-D* the dorsal segment of RLL, *RUL-A* the anterior segment of RUL, *n/a* not applicable.

For these two high-probability IMs cases, histopathological analysis revealed that the major histological subtype of all tumor samples was a acinar pattern (Table 2). One pair was a pGGN in the right upper lobe (RUL) and a mixed GGN (mGGN) in the right middle lobe (RML) of P10 (Fig. 3B). The other pair was two pGGNs of P16: one in the RUL and one in the RML (Fig. 3C). However, the conclusions of the nature of the nodules in these two cases were drawn from the images scanned on 5 mm-thick CT sections, making difficult to understand if the ‘GGN’ was a true GGN, since they might be evaluated as solid nodules on 1 mm thin CT sections^30^. In addition, postoperative progression-free survival (PFS) and overall survival (OS) of these two patients have not been reached. Thus, these two cases were diagnosed as MPs.

## Discussion

Due to the prevalence of MLCs, the etiology, diagnosis, staging, treatment, and prognosis of lung cancer aroused more attention in clinical practice, especially the distinction between MP and IM^4,12–14,19,31,32^. MLCs refer to multiple lung lesions from one side or two sides of the same patient, within the context of identical genetic background and exposure history. In order to improve the accuracy on distinguishing the origins of multiple lesions in patients with MLCs, three major lung cancer research institutes proposed and revised some diagnostic criteria, but unified standards are still lacking^6^.

Recent studies have pointed out that large panel next-generation sequencing assys can be utilized not only to guide targeted therapies, but also to determine the clonal relationships among MLCs^18,29^. Unlike conventional small gene panels, large panel next-generation sequencing assys can avoid the limitations of non-information in quite a few cases. A previous study on 76 tumor pairs from sixty patients have shown the utility of the large-scale gene assay (341–468 gene) for assessment of tumor clonal relationships^29^, but Asian populations were unrepresented in this cohort. More recently, the 464 gene panel have been applied to solve the problem of separating MPs from IMs^18^. The analysis involved 40 tumors in 16 patients, but in addition to lung tissues, tumor samples from breast, liver, thyroid, and mouth were also included. Therefore, it is necessary to conduct research from different perspectives and collect more samples from diverse populations to support the application of LSTS in clinical setting.

In this study, we performed 520 gene LSTS on 25 tumor pairs from 16 patients. Despite the shared germline mutation and environmental burden, paired tumors from 15 patients had distinct genomic profiles, including three tumors from P10 and two tumors from P16. As with the clinical diagnosis, they were all classified as MPs, although there were two patients (P2, P8) with lymph node metastases (Table 1). For tumor pairs classified as IMs, there were some shared variations, ranging from 2 to 6. Among them, one tumor pair with different histological subtypes from P5 were highly consistent in the somatic mutational profile. Considering that lung cancer usually displays a series of histological subtypes, different lesions often share overlapping histological features, which suggests that the morphology of MLC might not always be completely different. Therefore, the histological similarity between different tumors might be suggestive rather than conclusive^22,24^. In addition, lymph node metastases revealed that the two lesions were generated from the same clone. The above results suggested that this tumor pair originated from a common ancestor and the clinical diagnosis should be revised. Considering that pathologic, clinical and radiologic inferences depend on a doctor’s experience and thus, it may be subjective, LSTS can be an effective and objective complementary tool in clinic practice.

Another issue that deserves special attention is the multiple GGNs, which mainly include pGGNs and mGGNs. It is generally accepted that multiple GGNs in patients come out from different lineages, meaning that hematogenous metastasis do not happen to GGNs^5,30,31,33^. However, the possibility that a small number of GGNs come from the same ancestor cannot be excluded, which can be explained by the air space theory^25,34–36^. In our cohort, the definite IM case (a pGGN and a solid nodule) shared multiple mutations, providing evidence that a small portion of GGNs might be the result of early metastasis.

Besides, the recommendations given by the Fleishner Society state that lesions could be wrongly diagnosed as mGGNs on thick CT sections (typically 5 mm) when they are actually solid^30^. Nevertheless, the two high-probability IMs cases (P10 and P16) in this study had CT data from 5 mm-thick sections, which could not accurately determine whether the lesions were GGNs. Considering this issue and the LSTS results of these two tumor pairs, the possibility that these two nodules in P10 located in RUL and RML were descended from the same ancestor cannot be excluded. P16 confirmed the same. It does emphasize the importance of using contiguous thin CT sections (1 mm) to verify that the lesions are true GGNs. Moreover, no other mutations were shared in the remaining samples from P10 and P16, indicating that the other three lesions in P10 and the other two lesions in P16 were independent primary tumors. Above all, LSTS results revealed that both primary and metastatic cancers might exist in those patients, which is the most overlooked aspect of clinical work. However, since neither patient reached PFS and OS, follow-up is needed to confirm this conclusion.

The guidelines by the Fleishner Society also point out that GGNs progress slowly to various degrees, but the oncogenetic molecular mechanisms remain elusive^30^. Notably, the latest research suggested that the pre-cancerous unstable CNV with potentially genetic susceptibility may promote the development of driver mutations and independent synchronous multiple GGNs^34^. However, it remains unclear how the genetic map changes in the diversification from a GGN to a solid nodule, what factors influence this process and how to analyze it. Compared with LSTS, comprehensive genomic profiling at the whole exome or genome-level may be much more helpful to address this problem.

Additionally, some other challenges remain for a wider use of LSTS, including strict technological requirements, high cost, and long turnaround period. Moreover, because of the existence of negative results and common mutation sharing, its utility in making a distinction between MP and IM is limited^20^. In this study, no mutations were detected in one patient, which means that the 520 gene panel was uninformative in 2.8% of the cases. However, our work demonstrated that this problem can be solved by integrating clinical, radiologic and histological data.

There were two limitations to our study. Firstly, the limited cases could induce a slight bias in the genomic comparative analysis. We will further verify our conclusions by integrating public statistics and collecting more clinical cases. Secondly, we would have provided detailed histologic assessment of the study cases ideally, particularly of GGO or presumable AIS/lepidic cases. Some previous studies have provided more complete histological information for more cases. The results of those studies and ours are comparable in that they show that standard histopathological methods are sufficient in most cases, but have obvious limitations in the recognition of MLCs. In order to further probe into the development mechanism of MLCs, we intend to analyze the immune repertoire of MLCs, integrate multi-omics data, and conceive a more systematical and holistical approach to avoid the above problems.

Our findings have not only demonstrated the effectiveness of LSTS in distinguishing MP and IM but also provided evidence for the air space theory and the early metastasis of GGNs. The LSTS in this study was significantly increasing the diagnostic accuracy of patients with MLCs and can be used for guiding clinical treatments and achieving surveillance throughout the course of the therapy. In order to offer the best clinical management for patients with MLCs, larger targeted next-generation sequencing panels should be brought into the clinical detection in order to offer the best clinical management for patients with MLCs.

## Methods

### Patients selection and sample preparation

A total of 16 patients diagnosed with MPs at the First Affiliated Hospital of Chongqing Medical University between August 2016 and June 2018 were enrolled in this study, and 36 tissue samples were collected after surgery. This study was performed with the approval of the Institutional Review Board and the consent provided by each patient. Clinical, pathological, and radiological data of each patient were retrospectively collected from the electronic medical record system. The histopathological type of the tumor was evaluated by the analysis of the specimans performed by two independent pathologists.

### Tissue DNA extraction

The DNA was extracted from formalin-fixed paraffin-embedded (FFPE) tissues by QIAamp DNA FFPE tissue kit (Qiagen) according to the manufacturer’s protocol and then the purified DNA concentration was measured by Qubit dsDNA assay.

### Capture-based targeted DNA sequencing

Genetic profiles of all tissue samples were assessed by performing capture-based targeted deep sequencing using the OncoScreen Plus panel (Burning Rock Biotech Ltd., Guangzhou, China), including the entire exon regions of 312 genes and the hotspot mutation regions (exons, introns, and promoter regions) of 208 genes. In addition, 16 fusion genes were detected. The 520 cancer related genes used in the panel are listed in Table S2. A wide spectrum of mutation types was found, including large genomic rearrangement, copy number variation (CNV), insertion, deletion, stop-gain, frameshift, splice variant, missense, and mutations.

### Statistical analysis

NGS-based analysis were submitted to Burning Rock Biotech, a College of American Pathologists (CAP)-accredited/Clinical Laboratory Improvement Amendments (CLIA)-certified clinical laboratory and processed using optimized protocols as previously described^21^. The FASTQ format sequencing data were mapped to the human genome (hg19) using a BWA aligner 0.7.10^37^. With the use of GATK 3.2, MuTect and VarScan, local alignment optimization, variant calling, and annotation were performed respectively. The copy number cut-off of 1.5 corresponds to copy number deletion and 2.64 for copy number amplifications^21^. Variants were filtered using the VarScan fpfilter pipeline, and loci with depth less than 100 were filtered out^38^. DNA translocation analysis was performed using Tophat2 and Factera1.4.3. After reading the depth of each region using the total reading and region size and correcting the GC bias using the LOESS algorithm, the genetic level CNV was evaluated using the t statistic. Pairing analysis was used to assess the patterns of somatic mutations and CNV in the same individual, and a total of 25 comparisons were performed.

### Ethical statement

1. This study was performed with the approval of the Institutional Review Board of the First Affiliated Hospital of Chongqing Medical University (No. 2020-124).
2. All patients signed an informed consent document for the publication of this manuscript and any accompanying images.
3. The authors are accountable for all aspects of the work in ensuring that questions related to the accuracy or integrity of any part of the work are appropriately investigated and resolved.
4. All experiments were performed in accordance with relevant guidelines and regulations.

## Supplementary information

Supplementary Tables.
