# Supplementary material for: Application of large-scale targeted sequencing to distinguish multiple lung primary tumors from intrapulmonary metastases
Source: Sci Rep. 2020 Nov 2;10:18840. doi: 10.1038/s41598-020-75935-4 (PMC7606457; doi:10.1038/s41598-020-75935-4)
Supplement: Supplementary file 1 — Supplementary Tables. [file 41598_2020_75935_MOESM1_ESM.docx]

**Application of** **large-scale targeted sequencing to distinguish multiple lung primary tumors from intrapulmonary metastases**

Jiaxin Duan ^1^, Mingjian Ge ^2^, Jian Peng ^1^, Yangli Zhang ^1^, Li Yang ^3^, Ting Wang ^3^, Tian Qin ^4^, Rui Yuan ^5^, Yuhong Zhang ^1, *^, Wei Cheng ^1, *^

^1^ The Center for Clinical Molecular Medical detection, The First Affiliated Hospital of Chongqing Medical University, Chongqing 400016, P.R. China

^2^ Department of Cardiothoracic Surgery, The First Affiliated Hospital of Chongqing Medical University, Chongqing 400016, P.R. China

^3^ Department of Respiratory and Critical Care Medicine, the First Affiliated Hospital of Chongqing Medical University, Chongqing 400016, P.R. China

^4^ Burning Rock Biotech, Guangzhou 510300, P.R. China

^5^ Key Laboratory of Biorheological Science and Technology of Ministry of Education, School of Bioengineering, Chongqing University, Chongqing 400044, P.R. China

* Corresponding. chengwei@hospital.cqmu.edu.cn (W. Cheng), zhangyh1963@126.com (Y. Zhang)

**Table S1.** Comprehensive data of patients with multiple lung cancers.

| ID | Age | Gender | Smoking status | Location | Size (cm) | CT (mm) | Radiological feature | Histology | Histological subtype | | | | | Node staging |
| --- | --- | --- | --- | --- | --- | --- | --- | --- | --- | --- | --- | --- | --- | --- |
|  |  |  |  |  |  |  |  |  | Acinar (%) | Solid (%) | Papillary (%) | Micro-papillary (%) | Adherent (%) |  |
| P1 | 59 | Male | Former smoker | RUL | 3*2 | n/a | n/a | IA | 30 | 70 | 0 | 0 | 0 | IA3/T1cN0M0 |
|  |  |  |  | LUL | d=1.5 | 0.6 | GGN | IA | >80 | 0 | <20 | 0 | 0 | IA2/T1bN0M0 |
| P2 | 52 | Male | Former smoker | RUL | 2.5*2.5 | n/a | n/a | IA | <35 | 0 | >60 | <5 | 0 | ⅢA/T1cN2bM0 |
|  |  |  |  | LUL | 6.5*6.5 | n/a | n/a | SC | n/a | n/a | n/a | n/a | n/a | ⅡB/T3N0M0 |
| P3 | 62 | Male | Former smoker | RLL | d=1.3 | 2 | Part-solid | IA | 100 | 0 | 0 | 0 | 0 | IB/T2aN0M0 |
|  |  |  |  | RUL | d=1.5 | 2 | Part-solid | IA | 100 | 0 | 0 | 0 | 0 | IB/T2aN0M0 |
| P4 | 64 | Male | Former smoker | RUL | 2.2*0.2*0.3 | 0.6 | GGN | IA | 100 | 0 | 0 | 0 | 0 | IA3/T1c(2)N0M0 |
|  |  |  |  | RML | d=1.5 | 0.6 | Solid | IA | 100 | 0 | 0 | 0 | 0 | IA3/T1c(2)N0M0 |
| P5 | 54 | Female | Non-smoker | LUL-I | 1.5*1.6 | 0.6 | GGN | IA | 10 | 0 | 75 | 5 | 10 | ⅢA/T1bN2bM0 |
|  |  |  |  | LUL-U | 1.9*1.5 | 0.6 | Solid | IA | 90 | 0 | 5 | 5 | 0 | ⅢA/T2aN2bM0 |
| P6 | 65 | Male | Non-smoker | RML | 2.5*2.2 | 5 | GGN | MIA | n/a | n/a | n/a | n/a | n/a | IA/T1aN0M0 |
|  |  |  |  | RUL | 2.5*2.2 | 5 | Part-solid | IA | 100 | 0 | 0 | 0 | 0 | IA/T1bN0M0 |
| P7 | 73 | Female | Non-smoker | LLL | d=0.8 | n/a | GGN | AIS | 100 | 0 | 0 | 0 | 0 | IA/T1aN0M0 |
|  |  |  |  | LUL | d=0.7 | n/a | Solid | IA | n/a | n/a | n/a | n/a | n/a | IA/T1aN0M0 |
| P8 | 70 | Female | Non-smoker | RUL | 3*2.5 | 0.9 | Solid | IA | >70 | 0 | <30 | 0 | 0 | ⅡA/T2aN1M0 |
|  |  |  |  | RLL | 1.5*2.0 | 0.9 | GGN | IA | 100 | 0 | 0 | 0 | 0 | IA/T1aN0M0 |
| P9 | 69 | Male | Non-smoker | LLL | 1*1 | 0.6 | Part-solid | IA | 40 | 0 | 10 | 0 | 50 | IA1/T1aN0M0 |
|  |  |  |  | RLL | d=3 | n/a | Solid | IA | n/a | n/a | n/a | n/a | n/a | IA3/T1cN0M0 |
| P10 | 58 | Male | Former smoker | RLL-D | n/a | 5 | GGN | IA | 100 | 0 | 0 | 0 | 0 | n/a |
|  |  |  |  | RML | 1.9*3.7 | 5 | Part-solid | IA | 100 | 0 | 0 | 0 | 0 | IB/T2aN0M0 |
|  |  |  |  | RUL-A | n/a | 5 | GGN | IA | 100 | 0 | 0 | 0 | 0 | n/a |
|  |  |  |  | RUL | n/a | 5 | GGN | IA | 100 | 0 | 0 | 0 | 0 | n/a |
| P11 | 53 | Female | Non-smoker | LUL | d=0.6 | 3 | GGN | MIA | n/a | n/a | n/a | n/a | n/a | IA1/T1aN0M0 |
|  |  |  |  | RML | d=1.2 | 3 | GGN | MIA | n/a | n/a | n/a | n/a | n/a | IA1/T1a(mi)N0M0 |
| P12 | 54 | Female | Non-smoker | RUL | d=1.5 | 5 | GGN | IA | 0 | 0 | 0 | 0 | 100 | IA3/T1cN0M0 |
|  |  |  |  | LUL | d=0.8 | 5 | GGN | IA | 0 | 0 | 0 | 0 | 100 | IA2/T1bN0M0 |
| P13 | 55 | Female | Non-smoker | RML | 2*2.5 | 5 | Part-solid | IA | 0 | 0 | 100 | 0 | 0 | IB/T2aN0M0 |
|  |  |  |  | RLL | n/a | 5 | n/a | IA | 0 | 0 | 100 | 0 | 0 | IA/T1aN0M0 |
|  |  |  |  | RLL-D | 2*1 | 5 | Part-solid | IA | n/a | n/a | n/a | n/a | n/a | IB/T2aN0M0 |
| P14 | 54 | Female | Non-smoker | RLL | 1.6*1.4 | 5 | Solid | IA | 0 | 0 | 100 | 0 | 0 | IA/T1aN0M0 |
|  |  |  |  | RUL | 1.7*1.8 | 5 | GGN | IA | 100 | 0 | 0 | 0 | 0 | IA/T1aN0M0 |
| P15 | 66 | Female | Non-smoker | RML | 2*2.5 | 5 | Solid | IA | >60 | <40 | 0 | 0 | 0 | IA1/T1aN0M0 |
|  |  |  |  | RUL | n/a | 5 | GGN | MIA | n/a | n/a | n/a | n/a | n/a | IB/T2aN0M0 |
| P16 | 61 | Female | Non-smoker | LUL | 0.8*0.7 | 5 | GGN | IA | 100 | 0 | 0 | 0 | 0 | IA1/T1aN0M0 |
|  |  |  |  | RML | d=1.5 | 5 | GGN | IA | 100 | 0 | 0 | 0 | 0 | IA1/T1aN0M0 |
|  |  |  |  | RUL | 1.6*1.6 | 5 | GGN | IA | 100 | 0 | 0 | 0 | 0 | IA2/T1bN0M0 |
| IA, Invasive Adenocarcinoma; AIS, Adenocarcinoma *in situ*; MIA, Minimal invasive adenocarcinoma; SC, Sarcomatous carcinoma; GGN, Ground-glass nodule; CT, Computed Tomography; LLL, left lower lobe; LUL, left upper lobe; RLL, right lower lobe; RML, right middle lobe; RUL, right upper lobe; LUL-U, the upper lingual segment of LUL; LUL-I, the inferior lingual segment of LUL; RLL-D, the dorsal segment of RLL; RUL-A, the anterior segment of RUL; d, diameter; n/a, not applicable. | | | | | | | | | | | | | | |

**Table S2.** Gene list of 520 cancer-related genes.

| The entire exon regions (312 genes) | | | | | | | | | | | | |
| --- | --- | --- | --- | --- | --- | --- | --- | --- | --- | --- | --- | --- |
| ABL1 | BCOR | CDKN1A | EMSY | FBXW7 | GRIN2A | KDM5A | MITF | NRAS | PPP2R2A | RPTOR | STK11 | FAM46C |
| AKT1 | BLM | CDKN1B | EP300 | FGF19 | GRM3 | KDM5C | MLH1 | NSD1 | PRDM1 | RUNX1 | SUFU | FANCF |
| AKT2 | BRAF | CDKN1C | EPHA3 | FGF3 | GSK3B | KDM6A | MLH3 | NTHL1 | PRKAR1A | SDHA | SYK | FOXL2 |
| AKT3 | BRCA1 | CDKN2A | EPHA5 | FGF4 | GSTM1 | KDR | MPL | NTRK1 | PRKDC | SDHB | TBX3 | GREM1 |
| ALK | BRCA2 | CDKN2B | EPHA7 | FGFR1 | GSTT1 | KEAP1 | MRE11A | NTRK2 | PTCH1 | SDHC | TERT | H3F3C |
| APC | BRD4 | CDKN2C | EPHB1 | FGFR2 | H3F3A | KIT | MSH2 | NTRK3 | PETN | SDHD | TET2 | HIST1H1C |
| AR | BRIP1 | CEBPA | ERBB2 | FGFR3 | HGF | KMT2A | MSH3 | NUP93 | PTPN11 | SETD2 | TGFBR2 | HIST1H2BD |
| ARAF | BTK | CHD1 | ERBB3 | FGFR4 | HNF1A | KMT2C | MSH6 | PALB2 | PTPRD | SF3B1 | TNFAIP3 | HIST1H3A |
| ARID1A | CARD11 | CHEK1 | ERBB4 | FH | HNF1B | KMT2D | MTOR | PARK2 | RAC1 | SLX4 | TNFRSF14 | HIST1H3B |
| ARID1B | CBFB | CHEK2 | ERCC1 | FLCN | HRAS | KRAS | MUTYH | PAX5 | RAD50 | SMAD2 | TNFSF11 | HIST1H3C |
| ARID2 | CBL | CIC | ERG | FLT1 | IDH1 | LATS1 | MYC | PBRM1 | RAD51 | SMAD3 | TOP1 | HIST1H3D |
| ASXL1 | CCND1 | CREBBP | ERRFI1 | FLT3 | IDH2 | LMO1 | MYCL | PDGFRA | RAD51B | SMAD4 | TP53 | HIST1H3E |
| ATM | CCND2 | CRKL | ESR1 | FLT4 | IGF1R | LRP1B | MYCN | PDGFRB | RAD51C | SMARCA4 | TP63 | HIST1H3G |
| ATR | CCND3 | CRLF2 | EZH2 | FOXO1 | IGF2 | MAP2K1 | MYD88 | PIK3CA | RAD51D | SMARCB1 | TSC1 | HIST1H3H |
| ATRX | CCNE1 | CSF1R | FAM175A | FOXP1 | IKBKE | MAP2K2 | NBN | PIK3CB | RAD52 | SMO | TSC2 | HIST1H3I |
| AURKA | CD274 | CTCF | FANCA | FUBP1 | IKZF1 | MAP2K4 | NF1 | PIK3CG | RAD54L | SOX9 | TSHR | HIST1H3J |
| AURKB | CD79A | CTNNB1 | FANCC | GALNT12 | IL7R | MAP3K1 | NF2 | PIK3R1 | RAF1 | SPEN | U2AF1 | HIST2H3D |
| AXIN1 | CD79B | CUL3 | FANCD2 | GATA1 | INHBA | MCL1 | NFE2L2 | PIK3R2 | RARA | SPOP | VEGFA | HIST3H3 |
| AXL | CDC73 | DAXX | FANCE | GATA2 | INPP4B | MDM2 | NFKBIA | PLCG2 | RB1 | SPTA1 | VHL | IRS1 |
| BAP1 | CDH1 | DDR2 | FANCG | GATA3 | IRF4 | MDM4 | NKX2-1 | PMS1 | RBM10 | SRC | WRN | JUN |
| BARD1 | CDK12 | DICER1 | FANCI | GLI1 | IRS2 | MED12 | NOTCH1 | PMS2 | RET | SRSF2 | WT1 | POM121L12 |
| BCL2 | CDK4 | DNMT3A | FANCL | GNA11 | JAK1 | MEF2B | NOTCH2 | POLD1 | RICTOR | STAG2 | XPO1 | SOCS1 |
| BCL2L1 | CDK6 | DOT1L | FAT1 | GNAQ | JAK2 | MEN1 | NOTCH3 | POLE | RNF43 | STAT3 | XRCC2 | SOX2 |
| BCL6 | CDK8 | EGFR | FAT3 | GNAS | JAK3 | MET | NPM1 | PPP2R1A | ROS1 | STAT5B | AMER1 | TERC |
| The hotspot mutation regions (exons, introns, and promoter regions) (208 genes) | | | | | | | | | | | | |
| ABL2 | CHD2 | FGF12 | KAT6A | PAK3 | RECQL4 | TRAF7 | CD276 | ERCC4 | HSD3B1 | NEGR1 | PRSS8 | SUZ12 |
| ACVR1 | CHD4 | FGF14 | KEL | PAK7 | RHOA | TRRAP | CENPA | ERCC5 | ICOSLG | NKX3-1 | RAB35 | TACC3 |
| ACVR1B | CHUK | FRS2 | LATS2 | PARP2 | RPS6KA4 | TES1 | CRBN | FANCM | ID3 | PARP1 | RAD21 | TET1 |
| ADGRA2 | CSF3R | FYN | MAGI2 | PARP3 | RPS6KB2 | ZFHX3 | CTLA4 | FAS | IFNGR1 | PDCD1 | RANBP2 | TGFBR1 |
| ALOX12B | CUL4A | GABRA6 | MAP3K13 | PARP4 | RUNX1T1 | ZNF217 | CTNNA1 | FGF10 | IL10 | PDCD1LG2 | REL | TIRARP |
| ANKRD11 | CUL4B | GATA6 | MAPK3 | PGR | SH2B3 | ZNF703 | CXCR4 | FGF23 | INHA | PDK1 | RFWD2 | TMEM127 |
| ARID5B | CYLD | HDAC1 | MAX | PIK3C2G | SLIT2 | ZNRF3 | CYP17A1 | FGF6 | KLF4 | PDPK1 | RHEB | TOP2A |
| ASXL2 | DNMT1 | HDAC2 | MDC1 | PIK3CD | SMARCD1 | APCDD1 | DCUN1D1 | FGF7 | KLHL6 | PHOX2B | RIT1 | VEGFB |
| ATF1 | DNMT3B | HDAC4 | MST1 | PIM1 | SNCAIP | ARFRP1 | DIS3 | FOXA1 | LYN | PIK3C2B | RPA1 | VEGFC |
| AXIN2 | EIF4E | HIST1H3F | MST1R | PPP6C | STAT5A | BACH1 | DNAJB1 | GATA4 | LZTR1 | PIK3C3 | RYBP | VTCN1 |
| B2M | ELOC | HILA·A | NCOR1 | PREX2 | STK40 | BBC3 | E2F3 | GID4 | MALT1 | PIK3R3 | SDHAF2 | WISP3 |
| BCORL1 | EPCAM | HSP90AA1 | NEB | PTK2 | TAF1 | BCL10 | EED | GNA13 | MAP3K14 | PLK2 | SH2D1A | XIAP |
| BCR | EPHA22 | IGF1 | NOTCH4 | PTPRS | TCF3 | BCL2L11 | EGFL7 | GPS2 | MAPK1 | PMAIP1 | SHQ1 | XRCC3 |
| BIRC3 | ERCC2 | INPP4A | NR4A3 | PTPRT | TCF7L2 | BCL2L2 | EIF1AX | H3F3B | MGA | PNRC1 | SOX10 | YAP1 |
| BMPR1A | EWSR1 | INSR | NRG1 | QKI | TMPRSS2 | BTG1 | EIF4A2 | HIST2H3C | MYOD1 | PPM1D | SOX17 | ZBTB2 |
| CASP8 | FCGR2B | IRF2 | PAK1 | RASA1 | TRAF2 | CALR | ERCC3 | HOXB13 | NCOA3 | PRKCI | STAT4 | ZRSR2 |
| Fusion genes (16 genes) | | | | | | | | | | | | |
| ALK | BCR | BRAF | ERBB4 | EWSR1 | FGFR1 | FGFR2 | FGFR3 | MET | NRG1 | NTRK1 | NTRK2 | NTRK3 |
| RET | ROS1 | TMPRSS2 |  |  |  |  |  |  |  |  |  |  |

**Table S3.** List of all variations detected by target sequencing of 36 tumors from 16 patients.

| Sample_ID | Chrom | Position | Gene | HGVS_C | Description | Mutation_Type |
| --- | --- | --- | --- | --- | --- | --- |
| P1-LUL | 19 | 45916815 | *ERCC1* | c.962_963delinsCA | p.F321S | missense_variant |
| P1-LUL | 5 | 1272295 | *TERT* | c.2382+5G>T | c.2382+5G>T | splice_region_variant |
| P1-LUL | 7 | 2955019 | *CARD11* | c.2704-14del | c.2704-14del | intron_variant |
| P1-LUL | 17 | 7577551 | *TP53* | c.730G>T | p.G244C | missense_variant |
| P1-LUL | 1 | 8073390 | *ERRFI1* | c.1269C>G | p.I423M | missense_variant |
| P1-LUL | 20 | 9520208 | *PAK5* | c.2061G>A | p.Q687= | synonymous_variant |
| P1-LUL | 18 | 10471781 | *APCDD1* | c.497A>T | p.Q166L | missense_variant |
| P1-LUL | 1 | 11184710 | *MTOR* | c.6527-20G>C | c.6527-20G>C | intron_variant |
| P1-LUL | 1 | 16262005 | *SPEN* | c.9270G>C | p.L3090= | synonymous_variant |
| P1-LUL | 12 | 18641414 | *PIK3C2G* | c.2536T>C | p.F846L | missense_variant |
| P1-LUL | 12 | 25398281 | *KRAS* | c.38G>A | p.G13D | missense_variant |
| P1-LUL | 17 | 29562934 | *NF1* | c.3871del | p.V1291fs | frameshift_variant |
| P1-LUL | 17 | 29667580 | *NF1* | c.6916G>T | p.E2306* | stop_gained |
| P1-LUL | 3 | 37067437 | *MLH1* | c.1348G>T | p.D450Y | missense_variant |
| P1-LUL | 8 | 37555465 | *ZNF703* | c.1046G>T | p.G349V | missense_variant |
| P1-LUL | 20 | 40747148 | *PTPRT* | c.2942-8G>T | c.2942-8G>T | intron_variant |
| P1-LUL | 20 | 41514447 | *PTPRT* | c.214G>T | p.G72* | stop_gained |
| P1-LUL | 7 | 41729505 | *INHBA* | c.1024C>A | p.P342T | missense_variant |
| P1-LUL | 7 | 41729510 | *INHBA* | c.1019T>A | p.I340N | missense_variant |
| P1-LUL | 1 | 43804251 | *MPL* | c.251T>G | p.M84R | missense_variant |
| P1-LUL | 7 | 55270256 | *EGFR* | c.3209G>T | p.S1070I | missense_variant |
| P1-LUL | 4 | 55565774 | *KIT* | c.620-20del | c.620-20del | intron_variant |
| P1-LUL | 2 | 61719349 | *XPO1* | c.1724-16A>G | c.1724-16A>G | intron_variant |
| P1-LUL | 11 | 64137194 | *RPS6KA4* | c.1626G>T | p.T542= | synonymous_variant |
| P1-LUL | 11 | 64137687 | *RPS6KA4* | c.1798-10A>T | c.1798-10A>T | intron_variant |
| P1-LUL | 11 | 64573720 | *MEN1* | c.1048G>T | p.A350S | missense_variant |
| P1-LUL | 7 | 81336627 | *HGF* | c.1595C>A | p.A532E | missense_variant |
| P1-LUL | 15 | 88483855 | *NTRK3* | c.1715A>G | p.K572R | splice_region_variant |
| P1-LUL | X | 100614272 | *BTK* | c.894+9G>T | c.894+9G>T | intron_variant |
| P1-LUL | 2 | 109382484 | *RANBP2* | c.5489A>T | p.H1830L | missense_variant |
| P1-LUL | 1 | 158617412 | *SPTA1* | c.3812_3813delinsAT | p.W1271Y | missense_variant |
| P1-LUL | 1 | 158637781 | *SPTA1* | c.1905C>A | p.T635= | synonymous_variant |
| P1-LUL | 1 | 158644184 | *SPTA1* | c.1285G>T | p.A429S | missense_variant |
| P1-LUL | 1 | 158645971 | *SPTA1* | c.1072C>A | p.L358M | missense_variant |
| P1-LUL | 2 | 212989466 | *ERBB4* | c.234+11G>T | c.234+11G>T | intron_variant |
| P1-RUL | 19 | 1207032 | *STK11* | c.122del | p.K41fs | frameshift_variant |
| P1-RUL | 17 | 7574000 | *TP53* | c.1027G>T | p.E343* | stop_gained |
| P1-RUL | 17 | 7579503 | *TP53* | c.184G>T | p.E62* | stop_gained |
| P1-RUL | 10 | 8111571 | *GATA3* | c.1050+10C>G | c.1050+10C>G | intron_variant |
| P1-RUL | 20 | 9543619 | *PAK5* | c.1535G>T | p.S512I | missense_variant |
| P1-RUL | 3 | 10081516 | *FANCD2* | c.682G>T | p.G228W | missense_variant |
| P1-RUL | 17 | 29662044 | *NF1* | c.5938G>T | p.G1980W | missense_variant |
| P1-RUL | 17 | 41247958 | *BRCA1* | c.594-19C>T | c.594-19C>T | intron_variant |
| P1-RUL | X | 47040931 | *RBM10* | c.1656G>A | p.G552= | synonymous_variant |
| P1-RUL | 13 | 48947572 | *RB1* | c.1159A>G | p.M387V | missense_variant |
| P1-RUL | 7 | 53104214 | *POM121L12* | c.850G>T | p.A284S | missense_variant |
| P1-RUL | X | 53228000 | *KDM5C* | c.2314A>T | p.T772S | missense_variant |
| P1-RUL | 4 | 55138664 | *PDGFRA* | c.1341G>A | p.W447* | stop_gained |
| P1-RUL | 4 | 55955978 | *KDR* | c.3193-9C>G | c.3193-9C>G | splice_region_variant |
| P1-RUL | 20 | 57428516 | *GNAS* | c.196G>T | p.E66* | stop_gained |
| P1-RUL | 4 | 66233104 | *EPHA5* | c.1898A>G | p.E633G | missense_variant |
| P1-RUL | 8 | 69104611 | *PREX2* | c.4455G>T | p.L1485= | synonymous_variant |
| P1-RUL | 7 | 81346588 | *HGF* | c.1364del | p.G455fs | frameshift_variant |
| P1-RUL | 15 | 88472619 | *NTRK3* | c.1936C>A | p.Q646K | missense_variant |
| P1-RUL | 3 | 89445070 | *EPHA3* | c.1390A>G | p.N464D | missense_variant |
| P1-RUL | 11 | 92592463 | *FAT3* | c.11633G>A | p.R3878K | missense_variant |
| P1-RUL | 6 | 93979215 | *EPHA7* | c.1613C>A | p.A538D | missense_variant |
| P1-RUL | 6 | 94120643 | *EPHA7* | c.408G>C | p.R136S | missense_variant |
| P1-RUL | 7 | 106520039 | *PIK3CG* | c.2467C>A | p.L823I | missense_variant |
| P1-RUL | 7 | 106545618 | *PIK3CG* | c.3095C>T | p.S1032F | missense_variant |
| P1-RUL | 12 | 121416817 | *HNF1A* | c.246G>T | p.T82= | synonymous_variant |
| P1-RUL | 2 | 141528486 | *LRP1B* | c.5590G>T | p.G1864* | stop_gained |
| P1-RUL | 2 | 141816634 | *LRP1B* | c.1237-11C>A | c.1237-11C>A | intron_variant |
| P1-RUL | 2 | 141986994 | *LRP1B* | c.608C>A | p.P203Q | missense_variant |
| P1-RUL | 2 | 142567875 | *LRP1B* | c.178G>A | p.D60N | missense_variant |
| P1-RUL | 7 | 142658073 | *KEL* | c.342C>G | p.T114= | synonymous_variant |
| P1-RUL | 1 | 158585118 | *SPTA1* | c.6676G>C | p.A2226P | missense_variant |
| P1-RUL | 1 | 158637680 | *SPTA1* | c.2006G>T | p.W669L | missense_variant |
| P1-RUL | 5 | 170817072 | *NPM1* | c.76G>A | p.D26N | missense_variant |
| P1-RUL | 4 | 187531034 | *FAT1* | c.9989A>G | p.D3330G | missense_variant |
| P1-RUL | 3 | 192053203 | *FGF12* | c.361G>T | p.V121L | missense_variant |
| P1-RUL | 12p12.1 | 12p12.1 | *KRAS* | cn_amp | cn_amp | cn_amp |
| P1-RUL | 18q21.1 | 18q21.1 | *SMAD2* | cn_amp | cn_amp | cn_amp |
| P2-LUL | 17 | 7578190 | *TP53* | c.659A>G | p.Y220C | missense_variant |
| P2-LUL | 9 | 8460475 | *PTPRD* | c.3811G>A | p.V1271I | missense_variant |
| P2-LUL | 9 | 21971194 | *CDKN2A* | c.164G>A | p.G55D | missense_variant |
| P2-LUL | 9 | 21974759 | *CDKN2A* | c.67_68delinsTT | p.G23F | missense_variant |
| P2-LUL | 17 | 29553451 | *NF1* | c.2002-2A>G | c.2002-2A>G | splice_acceptor_variant |
| P2-LUL | 8 | 48701554 | *PRKDC* | c.10811dup | p.N3604fs | frameshift_variant |
| P2-LUL | 5 | 86685182 | *RASA1* | c.2926-25_2937del | p.N976_L980del | splice_acceptor_variant |
| P2-LUL | 11 | 100920688 | *PGR* | c.2460T>C | p.C820= | synonymous_variant |
| P2-LUL | 9 | 133761062 | *ABL1* | c.3385C>T | p.Q1129* | stop_gained |
| P2-LUL | 7 | 151970808 | *KMT2C* | c.994G>T | p.D332Y | missense_variant |
| P2-LUL | 3 | 178936091 | *PIK3CA* | c.1633G>A | p.E545K | missense_variant |
| P2-LUL | 5p13.1 | 5p13.1 | *RICTOR* | cn_amp | cn_amp | cn_amp |
| P2-LUL | 5p13.2 | 5p13.2 | *IL7R* | cn_amp | cn_amp | cn_amp |
| P2-LUL | 5p15.33 | 5p15.33 | *SDHA* | cn_amp | cn_amp | cn_amp |
| P2-LUL | 5p15.33 | 5p15.33 | *TERT* | cn_amp | cn_amp | cn_amp |
| P2-LUL | 7p11.2 | 7p11.2 | *EGFR* | cn_amp | cn_amp | cn_amp |
| P2-LUL | 7p12.2 | 7p12.2 | *IKZF1* | cn_amp | cn_amp | cn_amp |
| P2-LUL | 7p22.2 | 7p22.2 | *CARD11* | cn_amp | cn_amp | cn_amp |
| P2-RUL | 19 | 2216445 | *DOT1L* | c.2091del | p.K698fs | frameshift_variant |
| P2-RUL | 16 | 3633312 | *SLX4* | c.4939G>A | p.A1647T | missense_variant |
| P2-RUL | 17 | 7574010 | *TP53* | c.1013_1016del | p.F338fs | frameshift_variant |
| P2-RUL | 18 | 10471659 | *APCDD1* | c.375G>T | p.R125= | synonymous_variant |
| P2-RUL | 12 | 18443803 | *PIK3C2G* | c.776A>T | p.Y259F | missense_variant |
| P2-RUL | 12 | 18793377 | *PIK3C2G* | c.4197C>G | p.G1399= | synonymous_variant |
| P2-RUL | 6 | 26031949 | *HIST1H3B* | c.340C>A | p.H114N | missense_variant |
| P2-RUL | 2 | 29443563 | *ALK* | c.3645+9G>A | c.3645+9G>A | intron_variant |
| P2-RUL | 8 | 30999016 | *WRN* | c.3038G>T | p.S1013I | missense_variant |
| P2-RUL | 20 | 40710512 | *PTPRT* | c.4329+10C>A | c.4329+10C>A | intron_variant |
| P2-RUL | 8 | 41906112 | *KAT6A* | c.384G>T | p.Q128H | missense_variant |
| P2-RUL | X | 47028880 | *RBM10* | c.379G>T | p.E127* | stop_gained |
| P2-RUL | 8 | 48817434 | *PRKDC* | c.3037A>G | p.I1013V | missense_variant |
| P2-RUL | 4 | 55156627 | *PDGFRA* | c.3028C>A | p.Q1010K | missense_variant |
| P2-RUL | 12 | 56482382 | *ERBB3* | c.930G>C | p.M310I | missense_variant |
| P2-RUL | 1 | 65339158 | *JAK1* | c.378G>C | p.V126= | synonymous_variant |
| P2-RUL | 4 | 66361195 | *EPHA5* | c.977G>T | p.S326I | missense_variant |
| P2-RUL | X | 66931547 | *AR* | c.2173+16G>C | c.2173+16G>C | intron_variant |
| P2-RUL | 15 | 88420200 | *NTRK3* | c.2486A>G | p.K829R | missense_variant |
| P2-RUL | 3 | 89176388 | *EPHA3* | c.118G>T | p.G40W | missense_variant |
| P2-RUL | 11 | 92088165 | *FAT3* | c.2887C>T | p.P963S | missense_variant |
| P2-RUL | 11 | 92533428 | *FAT3* | c.7249C>A | p.Q2417K | missense_variant |
| P2-RUL | 2 | 109381244 | *RANBP2* | c.4249G>C | p.G1417R | missense_variant |
| P2-RUL | X | 110406816 | *PAK3* | c.735C>A | p.S245= | synonymous_variant |
| P2-RUL | 12 | 115117788 | *TBX3* | c.658-11T>C | c.658-11T>C | splice_region_variant |
| P2-RUL | 2 | 141459844 | *LRP1B* | c.6168G>T | p.W2056C | missense_variant |
| P2-RUL | 2 | 141473522 | *LRP1B* | c.6023+20A>G | c.6023+20A>G | intron_variant |
| P2-RUL | 2 | 141526783 | *LRP1B* | c.5757A>T | p.A1919= | splice_region_variant |
| P2-RUL | 2 | 141643695 | *LRP1B* | c.3964+12A>T | c.3964+12A>T | intron_variant |
| P2-RUL | 2 | 141747194 | *LRP1B* | c.2677T>C | p.C893R | missense_variant |
| P2-RUL | 1 | 150550742 | *MCL1* | c.914G>T | p.W305L | missense_variant |
| P2-RUL | 4 | 153249521 | *FBXW7* | c.1256del | p.G419fs | frameshift_variant |
| P2-RUL | 1 | 158609691 | *SPTA1* | c.4844G>T | p.S1615I | missense_variant |
| P2-RUL | 2 | 220440130 | *INHA* | c.983G>T | p.C328F | missense_variant |
| P2-RUL | 1 | 228612975 | *HIST3H3* | c.52C>T | p.R18C | missense_variant |
| P2-RUL | 6p21.1 | 6p21.1 | *CCND3* | cn_amp | cn_amp | cn_amp |
| P2-RUL | 6p21.1 | 6p21.1 | *VEGFA* | cn_amp | cn_amp | cn_amp |
| P2-RUL | 6p21.2 | 6p21.2 | *CDKN1A* | cn_amp | cn_amp | cn_amp |
| P2-RUL | 6p21.2 | 6p21.2 | *PIM1* | cn_amp | cn_amp | cn_amp |
| P2-RUL | 6p21.32 | 6p21.32 | *DAXX* | cn_amp | cn_amp | cn_amp |
| P2-RUL | 7p11.2 | 7p11.2 | *EGFR* | cn_amp | cn_amp | cn_amp |
| P3-RLL | 12 | 1022565 | *RAD52* | c.1249C>A | p.P417T | missense_variant |
| P3-RLL | 12 | 1022624 | *RAD52* | c.1196-6C>T | c.1196-6C>T | intron_variant |
| P3-RLL | 1 | 16254819 | *SPEN* | c.2084A>G | p.Y695C | missense_variant |
| P3-RLL | 18 | 19751651 | *GATA6* | c.546G>A | p.A182= | synonymous_variant |
| P3-RLL | 6 | 20490573 | *E2F3* | c.1310G>A | p.S437N | missense_variant |
| P3-RLL | 16 | 23619318 | *PALB2* | c.3216dup | p.V1073fs | frameshift_variant |
| P3-RLL | 8 | 30945363 | *WRN* | c.1503G>A | p.L501= | synonymous_variant |
| P3-RLL | 20 | 39728715 | *TOP1* | c.997_1031del | p.K333fs | frameshift_variant |
| P3-RLL | 17 | 40498657 | *STAT3* | c.203A>G | p.Y68C | missense_variant |
| P3-RLL | 3 | 47163109 | *SETD2* | c.3017A>G | p.N1006S | missense_variant |
| P3-RLL | 8 | 48734175 | *PRKDC* | c.9098C>T | p.P3033L | missense_variant |
| P3-RLL | 7 | 55259515 | *EGFR* | c.2573T>G | p.L858R | missense_variant |
| P3-RLL | 14 | 75514927 | *MLH3* | c.1432G>A | p.A478T | missense_variant |
| P3-RLL | 15 | 89850842 | *FANCI* | c.3592-2A>G | c.3592-2A>G | splice_acceptor_variant |
| P3-RLL | 15 | 89850848 | *FANCI* | c.3596A>G | p.K1199R | missense_variant |
| P3-RLL | X | 123179074 | *STAG2* | c.523T>C | p.F175L | missense_variant |
| P3-RLL | 14q11.2 | 14q11.2 | *PARP2* | cn_amp | cn_amp | cn_amp |
| P3-RLL | 14q13.2 | 14q13.2 | *NFKBIA* | cn_amp | cn_amp | cn_amp |
| P3-RLL | 14q13.3 | 14q13.3 | *NKX2-1* | cn_amp | cn_amp | cn_amp |
| P3-RLL | 20q12 | 20q12 | *PTPRT* | cn_amp | cn_amp | cn_amp |
| P3-RUL | 19 | 11169457 | *SMARCA4* | c.4630-7A>T | c.4630-7A>T | intron_variant |
| P3-RUL | 14 | 23776982 | *BCL2L2* | c.6G>T | p.A2= | synonymous_variant |
| P3-RUL | 12 | 25398285 | *KRAS* | c.34G>T | p.G12C | missense_variant |
| P3-RUL | 22 | 29121033 | *CHEK2* | c.524T>C | p.V175A | missense_variant |
| P3-RUL | 9 | 36882099 | *PAX5* | c.913del | p.R305fs | frameshift_variant |
| P3-RUL | 9 | 37015049 | *PAX5* | c.355C>A | p.L119M | missense_variant |
| P3-RUL | 20 | 40877382 | *PTPRT* | c.2314G>T | p.G772C | missense_variant |
| P3-RUL | X | 70344939 | *MED12* | c.2169G>C | p.G723= | synonymous_variant |
| P3-RUL | 13 | 110437829 | *IRS2* | c.572C>T | p.T191M | missense_variant |
| P3-RUL | 7 | 140453163 | *BRAF* | c.1772A>T | p.K591I | missense_variant |
| P3-RUL | 2 | 141816445 | *LRP1B* | c.1408+7G>A | c.1408+7G>A | intron_variant |
| P4-RML | 10 | 8100355 | *GATA3* | c.329C>A | p.S110Y | missense_variant |
| P4-RML | 12 | 25398284 | *KRAS* | c.35G>T | p.G12V | missense_variant |
| P4-RML | 22 | 41523519 | *EP300* | c.935A>T | p.Q312L | missense_variant |
| P4-RML | 13 | 48936942 | *RB1* | c.719-9C>G | c.719-9C>G | splice_region_variant |
| P4-RML | 1 | 78425921 | *FUBP1* | c.1524G>T | p.Q508H | missense_variant |
| P4-RML | 14 | 81609461 | *TSHR* | c.1059C>A | p.Y353* | stop_gained |
| P4-RML | 3 | 89480333 | *EPHA3* | c.2170G>T | p.V724L | missense_variant |
| P4-RML | 12 | 133219542 | *POLE* | c.4592C>T | p.S1531L | missense_variant |
| P4-RML | 4 | 187538243 | *FAT1* | c.8991G>C | p.T2997= | synonymous_variant |
| P4-RUL | 19 | 1220612 | *STK11* | c.630C>A | p.C210* | stop_gained |
| P4-RUL | 16 | 3645665 | *SLX4* | c.1954C>G | p.L652V | missense_variant |
| P4-RUL | 1 | 11190628 | *MTOR* | c.5571G>A | p.E1857= | synonymous_variant |
| P4-RUL | 2 | 29519797 | *ALK* | c.1774C>A | p.L592M | missense_variant |
| P4-RUL | 5 | 35861019 | *IL7R* | c.148G>T | p.G50* | stop_gained |
| P4-RUL | 17 | 37879583 | *ERBB2* | c.1958C>G | p.S653C | missense_variant |
| P4-RUL | 7 | 41729926 | *INHBA* | c.603G>A | p.R201= | synonymous_variant |
| P4-RUL | 19 | 50919053 | *POLD1* | c.2790C>T | p.A930= | synonymous_variant |
| P4-RUL | 12 | 57861294 | *GLI1* | c.1077+14C>T | c.1077+14C>T | intron_variant |
| P4-RUL | 15 | 66727483 | *MAP2K1* | c.199G>T | p.D67Y | missense_variant |
| P4-RUL | 9 | 101599419 | *GALNT12* | c.1201C>T | p.R401C | missense_variant |
| P4-RUL | 7 | 128852008 | *SMO* | c.2080C>A | p.P694T | missense_variant |
| P4-RUL | 1 | 156851276 | *NTRK1* | c.2125G>A | p.E709K | missense_variant |
| P4-RUL | 2 | 178098975 | *NFE2L2* | c.70T>C | p.W24R | missense_variant |
| P4-RUL | 4 | 187557981 | *FAT1* | c.3730C>G | p.Q1244E | missense_variant |
| P5-LUL-I | 18 | 48593519 | *SMAD4* | c.1271del | p.D424fs | frameshift_variant |
| P5-LUL-I | 13 | 49027146 | *RB1* | c.1713_1714insA | p.L572fs | frameshift_variant |
| P5-LUL-I | 13 | 49033822 | *RB1* | c.1961-2A>T | c.1961-2A>T | splice_acceptor_variant |
| P5-LUL-I | 7 | 53103815 | *POM121L12* | c.451T>A | p.W151R | missense_variant |
| P5-LUL-I | 7 | 55242464 | *EGFR* | c.2235_2249del | p.E746_A750del | disruptive_inframe_deletion |
| P5-LUL-I | 5 | 67591246 | *PIK3R1* | c.1746-2A>G | c.1746-2A>G | splice_acceptor_variant |
| P5-LUL-I | 11 | 76162947 | *EMSY* | c.116G>A | p.G39E | missense_variant |
| P5-LUL-I | 5p15.33 | 5p15.33 | *SDHA* | cn_amp | cn_amp | cn_amp |
| P5-LUL-I | 5p15.33 | 5p15.33 | *TERT* | cn_amp | cn_amp | cn_amp |
| P5-LUL-I | 7p11.2 | 7p11.2 | *EGFR* | cn_amp | cn_amp | cn_amp |
| P5-LUL-U | 17 | 7577539 | *TP53* | c.742C>T | p.R248W | missense_variant |
| P5-LUL-U | 18 | 48604665 | *SMAD4* | c.1487G>A | p.R496H | missense_variant |
| P5-LUL-U | 7 | 53103815 | *POM121L12* | c.451T>A | p.W151R | missense_variant |
| P5-LUL-U | 7 | 55242464 | *EGFR* | c.2235_2249del | p.E746_A750del | disruptive_inframe_deletion |
| P5-LUL-U | 11 | 76162947 | *EMSY* | c.116G>A | p.G39E | missense_variant |
| P5-LUL-U | 10 | 89717766 | *PTEN* | c.792dup | p.L265fs | frameshift_variant |
| P5-LUL-U | 11 | 108119672 | *ATM* | c.1078G>C | p.D360H | missense_variant |
| P5-LUL-U | 13q14.2 | 48881349-48955600(+) | *RB1* | exon2-17cn_del | exon2-17cn_del | large_genomic_rearrangement |
| P5-LUL-U | 5p15.33 | 5p15.33 | *SDHA* | cn_amp | cn_amp | cn_amp |
| P5-LUL-U | 5p15.33 | 5p15.33 | *TERT* | cn_amp | cn_amp | cn_amp |
| P5-LUL-U | 7p11.2 | 7p11.2 | *EGFR* | cn_amp | cn_amp | cn_amp |
| P5-LUL-U | 8q11.21 | 8q11.21 | *PRKDC* | cn_amp | cn_amp | cn_amp |
| P5-LUL-U | 8q13.2 | 8q13.2 | *PREX2* | cn_amp | cn_amp | cn_amp |
| P5-LUL-U | 8q21.3 | 8q21.3 | *NBN* | cn_amp | cn_amp | cn_amp |
| P5-LUL-U | 8q21.3 | 8q21.3 | *RUNX1T1* | cn_amp | cn_amp | cn_amp |
| P6-RML | 5 | 1271315 | *TERT* | c.2387C>G | p.S796C | missense_variant |
| P6-RML | 12 | 4398019 | *CCND2* | c.583G>T | p.A195S | missense_variant |
| P6-RML | 17 | 7577105 | *TP53* | c.833C>G | p.P278R | missense_variant |
| P6-RML | 17 | 15960863 | *NCOR1* | c.6357C>T | p.V2119= | synonymous_variant |
| P6-RML | 17 | 15960883 | *NCOR1* | c.6337C>T | p.Q2113* | stop_gained |
| P6-RML | 22 | 41566462 | *EP300* | c.4339A>G | p.I1447V | missense_variant |
| P6-RML | 7 | 55259515 | *EGFR* | c.2573T>G | p.L858R | missense_variant |
| P6-RML | 18 | 60795891 | *BCL2* | c.687C>G | p.C229W | missense_variant |
| P6-RML | 16 | 89347927 | *ANKRD11* | c.5023G>C | p.D1675H | missense_variant |
| P6-RML | 6 | 114264505 | *HDAC2* | c.1378+10G>T | c.1378+10G>T | intron_variant |
| P6-RML | 1 | 156851293 | *NTRK1* | c.2142T>A | p.R714= | synonymous_variant |
| P6-RML | 5 | 180048668 | *FLT4* | c.1894C>T | p.R632C | missense_variant |
| P6-RML | 14q13.2 | 14q13.2 | *NFKBIA* | cn_amp | cn_amp | cn_amp |
| P6-RML | 14q13.3 | 14q13.3 | *NKX2-1* | cn_amp | cn_amp | cn_amp |
| P6-RML | 5p13.1 | 5p13.1 | *RICTOR* | cn_amp | cn_amp | cn_amp |
| P6-RML | 5p15.33 | 5p15.33 | *TERT* | cn_amp | cn_amp | cn_amp |
| P6-RML | 7p11.2 | 7p11.2 | *EGFR* | cn_amp | cn_amp | cn_amp |
| P6-RUL | 7 | 140453154 | *BRAF* | c.1781A>G | p.D594G | missense_variant |
| P7-LLL | 17 | 7577128 | *TP53* | c.810T>G | p.F270L | missense_variant |
| P7-LLL | 13 | 48921999 | *RB1* | c.539C>A | p.S180* | stop_gained |
| P7-LLL | 13 | 48934180 | *RB1* | c.635T>G | p.L212R | missense_variant |
| P7-LLL | 13 | 48934196 | *RB1* | c.651_653delinsATG | p.Q217* | stop_gained |
| P7-LLL | 7 | 53103492 | *POM121L12* | c.128C>T | p.T43M | missense_variant |
| P7-LLL | 7 | 55242464 | *EGFR* | c.2235_2249del | p.E746_A750del | disruptive_inframe_deletion |
| P7-LLL | 6 | 162683582 | *PRKN* | c.387G>C | p.K129N | missense_variant |
| P7-LLL | 1 | 206648348 | *IKBKE* | c.358+11C>T | c.358+11C>T | intron_variant |
| P7-LLL | 12q13.3 | 12q13.3 | *GLI1* | cn_amp | cn_amp | cn_amp |
| P7-LLL | 12q14.1 | 12q14.1 | *CDK4* | cn_amp | cn_amp | cn_amp |
| P7-LLL | 2q33.1 | 2q33.1 | *CASP8* | cn_amp | cn_amp | cn_amp |
| P7-LLL | 5p15.33 | 5p15.33 | *SDHA* | cn_amp | cn_amp | cn_amp |
| P7-LLL | 5p15.33 | 5p15.33 | *TERT* | cn_amp | cn_amp | cn_amp |
| P7-LUL | X | 39933150 | *BCOR* | c.1449G>A | p.P483= | synonymous_variant |
| P7-LUL | 7 | 55259515 | *EGFR* | c.2573T>G | p.L858R | missense_variant |
| P7-LUL | 7 | 77885404 | *MAGI2* | c.1903C>T | p.Q635* | stop_gained |
| P7-LUL | 3 | 119562121 | *GSK3B* | c.1215A>T | p.T405= | synonymous_variant |
| P8-RLL | 12 | 442677 | *KDM5A* | c.1629C>T | p.N543= | synonymous_variant |
| P8-RLL | 17 | 7578526 | *TP53* | c.404G>T | p.C135F | missense_variant |
| P8-RLL | 1 | 11269521 | *MTOR* | c.3655-6T>G | c.3655-6T>G | intron_variant |
| P8-RLL | 3 | 52440365 | *BAP1* | c.687C>A | p.N229K | missense_variant |
| P8-RLL | 7 | 55259515 | *EGFR* | c.2573T>G | p.L858R | missense_variant |
| P8-RLL | 15 | 93524031 | *CHD2* | c.2877-14T>G | c.2877-14T>G | intron_variant |
| P8-RUL | 5 | 231033 | *SDHA* | c.813C>G | p.T271= | synonymous_variant |
| P8-RUL | 13 | 21555725 | *LATS2* | c.2545C>T | p.R849W | missense_variant |
| P8-RUL | 17 | 29533261 | *NF1* | c.1264G>T | p.A422S | missense_variant |
| P8-RUL | 3 | 47142964 | *SETD2* | c.4999C>T | p.Q1667* | stop_gained |
| P8-RUL | 3 | 47163353 | *SETD2* | c.2769_2772del | p.H923fs | frameshift_variant |
| P8-RUL | 7 | 55242444 | *EGFR* | c.2217_2234dup | p.K745_E746insIPVAIK | disruptive_inframe_insertion |
| P8-RUL | 11 | 108199839 | *ATM* | c.7181C>T | p.S2394L | missense_variant |
| P8-RUL | 3 | 128205076 | *GATA2* | c.365C>T | p.S122F | missense_variant |
| P8-RUL | 3 | 178936091 | *PIK3CA* | c.1633G>A | p.E545K | missense_variant |
| P9-LLL | 7 | 55241707 | *EGFR* | c.2155G>T | p.G719C | missense_variant |
| P9-LLL | 7 | 55249005 | *EGFR* | c.2303G>T | p.S768I | missense_variant |
| P9-LLL | X | 110439158 | *PAK3* | c.1307C>T | p.S436F | missense_variant |
| P9-LLL | 6 | 157517384 | *ARID1B* | c.3952_3967del | p.M1318fs | frameshift_variant |
| P9-LLL | 1 | 206648322 | *IKBKE* | c.343G>T | p.V115L | missense_variant |
| P9-LLL | 14q13.2 | 14q13.2 | *NFKBIA* | cn_amp | cn_amp | cn_amp |
| P9-LLL | 14q13.3 | 14q13.3 | *NKX2-1* | cn_amp | cn_amp | cn_amp |
| P9-LLL | 1q32.1 | 1q32.1 | *IKBKE* | cn_amp | cn_amp | cn_amp |
| P9-RLL | 17 | 29554328 | *NF1* | c.2325+19A>T | c.2325+19A>T | intron_variant |
| P9-RLL | 3 | 30691802 | *TGFBR2* | c.379C>G | p.H127D | missense_variant |
| P9-RLL | 7 | 55242465 | *EGFR* | c.2239_2248delinsC | p.L747_A750delinsP | disruptive_inframe_deletion |
| P9-RLL | 8 | 55371922 | *SOX17* | c.612C>T | p.R204= | synonymous_variant |
| P9-RLL | 3 | 70005599 | *MITF* | c.635-7C>T | c.635-7C>T | intron_variant |
| P9-RLL | 5 | 112176183 | *APC* | c.4895_4896dup | p.T1633fs | frameshift_variant |
| P10-RLL-D | 17 | 29661996 | *NF1* | c.5890G>T | p.E1964* | stop_gained |
| P10-RLL-D | 17 | 29664601 | *NF1* | c.6579+1G>T | c.6579+1G>T | splice_donor_variant |
| P10-RLL-D | 20 | 40733205 | *PTPRT* | c.3597+4T>A | c.3597+4T>A | intron_variant |
| P10-RML | 17 | 7578442 | *TP53* | c.488A>G | p.Y163C | missense_variant |
| P10-RML | 19 | 15292426 | *NOTCH3* | c.2753G>A | p.G918D | missense_variant |
| P10-RML | X | 47039859 | *RBM10* | c.1399_1415del | p.V467fs | frameshift_variant |
| P10-RML | 17 | 47688775 | *SPOP* | c.525C>T | p.T175= | synonymous_variant |
| P10-RML | 7 | 55242467 | *EGFR* | c.2240_2254del | p.L747_T751del | disruptive_inframe_deletion |
| P10-RML | 13 | 113899524 | *CUL4A* | c.1503G>A | p.S501= | synonymous_variant |
| P10-RML | 12q15 | 12q15 | *MDM2* | cn_amp | cn_amp | cn_amp |
| P10-RUL | X | 47039859 | *RBM10* | c.1399_1415del | p.V467fs | frameshift_variant |
| P10-RUL | 7 | 55242467 | *EGFR* | c.2240_2254del | p.L747_T751del | disruptive_inframe_deletion |
| P10-RUL-A | 9 | 8500908 | *PTPRD* | c.1974G>C | p.K658N | missense_variant |
| P10-RUL-A | 7 | 55259515 | *EGFR* | c.2573T>G | p.L858R | missense_variant |
| P10-RUL-A | 9 | 133760948 | *ABL1* | c.3271G>T | p.E1091* | stop_gained |
| P11-LUL | 16 | 2097827 | *NTHL1* | c.22G>C | p.G8R | missense_variant |
| P11-LUL | 19 | 14627416 | *DNAJB1* | c.654C>T | p.I218= | synonymous_variant |
| P11-LUL | 17 | 29585451 | *NF1* | c.4200G>A | p.P1400= | synonymous_variant |
| P11-LUL | 17 | 29661941 | *NF1* | c.5838dup | p.R1947fs | frameshift_variant |
| P11-LUL | 11 | 92535051 | *FAT3* | c.8872C>T | p.R2958C | missense_variant |
| P11-LUL | 1 | 150551388 | *MCL1* | c.619A>G | p.R207G | missense_variant |
| P11-RML | X | 47041715 | *RBM10* | c.2136dup | p.A713fs | frameshift_variant |
| P11-RML | 7 | 55259515 | *EGFR* | c.2573T>G | p.L858R | missense_variant |
| P11-RML | 5p15.33 | 5p15.33 | *TERT* | cn_amp | cn_amp | cn_amp |
| P12-LUL | X | 47032536 | *RBM10* | c.637C>T | p.Q213* | stop_gained |
| P12-LUL | 7 | 55259515 | *EGFR* | c.2573T>G | p.L858R | missense_variant |
| P12-RUL | 13 | 29041708 | *FLT1* | c.111A>G | p.K37= | synonymous_variant |
| P12-RUL | X | 47045686 | *RBM10* | c.2765del | p.G922fs | frameshift_variant |
| P12-RUL | 7 | 55259515 | *EGFR* | c.2573T>A | p.L858Q | missense_variant |
| P12-RUL | 7 | 55259524 | *EGFR* | c.2582T>A | p.L861Q | missense_variant |
| P12-RUL | 9 | 102595066 | *NR4A3* | c.1080T>A | p.F360L | missense_variant |
| P13-RLL | 7 | 55242465 | *EGFR* | c.2236_2250del | p.E746_A750del | conservative_inframe_deletion |
| P13-RLL | 5 | 112173846 | *APC* | c.2555T>A | p.L852* | stop_gained |
| P13-RML | 16 | 3778363 | *CREBBP* | c.6685G>C | p.G2229R | missense_variant |
| P13-RML | 17 | 7577535 | *TP53* | c.745del | p.R249fs | frameshift_variant |
| P13-RML | 7 | 55259515 | *EGFR* | c.2573T>G | p.L858R | missense_variant |
| P13-RML | 7 | 116412172 | *MET* | c.3028+129A>G | c.3028+129A>G | intron_variant |
| P14-RLL | 7 | 6043405 | *PMS2* | c.269C>A | p.S90Y | missense_variant |
| P14-RLL | 7 | 55242464 | *EGFR* | c.2235_2249del | p.E746_A750del | disruptive_inframe_deletion |
| P14-RLL | 3 | 128204602 | *GATA2* | c.839C>T | p.P280L | missense_variant |
| P14-RUL | X | 47041719 | *RBM10* | c.2139_2140delinsCT | p.A713* | stop_gained |
| P14-RUL | 7 | 55259515 | *EGFR* | c.2573T>G | p.L858R | missense_variant |
| P14-RUL | 7 | 142643377 | *KEL* | c.1231G>A | p.V411M | missense_variant |
| P15-RML | 17 | 7578461 | *TP53* | c.469G>T | p.V157F | missense_variant |
| P15-RML | 8 | 48839785 | *PRKDC* | c.2388G>C | p.L796= | synonymous_variant |
| P15-RML | 7 | 55259515 | *EGFR* | c.2573T>G | p.L858R | missense_variant |
| P15-RUL | X | 47044724 | *RBM10* | c.2321_2330del | p.T774fs | frameshift_variant |
| P15-RUL | 7 | 55259515 | *EGFR* | c.2573T>G | p.L858R | missense_variant |
| P15-RUL | 4 | 187524835 | *FAT1* | c.10845C>T | p.S3615= | synonymous_variant |
| P16-LUL | 7 | 55242464 | *EGFR* | c.2235_2249del | p.E746_A750del | disruptive_inframe_deletion |
| P16-RML | 7 | 55259515 | *EGFR* | c.2573T>G | p.L858R | missense_variant |
| P16-RML | X | 76937714 | *ATRX* | c.3034T>G | p.S1012A | missense_variant |
| P16-RUL | X | 47040653 | *RBM10* | c.1483G>T | p.E495* | stop_gained |
| P16-RUL | 7 | 55259515 | *EGFR* | c.2573T>G | p.L858R | missense_variant |
| P16-RUL | X | 76937714 | *ATRX* | c.3034T>G | p.S1012A | missense_variant |

**Table S4**. Summary of somatic alterations, microsatellite instability and tumor mutation burden detected in tumors from Patient1-16 by target sequencing.

| Sample | Missense | Synonymous | Stop gain | Intron | Splice Site | Frameshift | Deletion | Insertion | Rearrangement | Copy Number Amplification | MSI | TMB |
| --- | --- | --- | --- | --- | --- | --- | --- | --- | --- | --- | --- | --- |
| P1-LUL | 18 | 4 | 2 | 8 | 2 | 1 | 0 | 0 | 0 | 0 | MSS | 27.78 |
| P1-RUL | 21 | 4 | 5 | 3 | 1 | 2 | 0 | 0 | 0 | 2 | MSS | 28.57 |
| P2-LUL | 6 | 1 | 1 | 0 | 2 | 1 | 0 | 0 | 0 | 7 | MSS | 8.73 |
| P2-RUL | 20 | 4 | 1 | 5 | 2 | 3 | 0 | 0 | 0 | 6 | MSS | 27.78 |
| P3-RLL | 10 | 2 | 0 | 1 | 1 | 2 | 0 | 0 | 0 | 4 | MSS | 11.90 |
| P3-RUL | 6 | 2 | 0 | 2 | 0 | 1 | 0 | 0 | 0 | 0 | MSS | 8.73 |
| P4-RML | 6 | 1 | 1 | 0 | 1 | 0 | 0 | 0 | 0 | 0 | MSS | 7.14 |
| P4-RUL | 9 | 3 | 2 | 1 | 0 | 0 | 0 | 0 | 0 | 0 | MSS | 11.90 |
| P5-LUL-I | 2 | 0 | 0 | 0 | 2 | 2 | 1 | 0 | 0 | 3 | MSS | 4.76 |
| P5-LUL-U | 5 | 0 | 0 | 0 | 0 | 1 | 1 | 0 | 1 | 7 | MSS | 4.76 |
| P6-RML | 8 | 2 | 1 | 1 | 0 | 0 | 0 | 0 | 0 | 5 | MSS | 8.73 |
| P6-RUL | 1 | 0 | 0 | 0 | 0 | 0 | 0 | 0 | 0 | 0 | MSS | 0.79 |
| P7-LLL | 4 | 0 | 2 | 1 | 0 | 0 | 1 | 0 | 0 | 5 | MSS | 5.56 |
| P7-LUL | 1 | 2 | 1 | 0 | 0 | 0 | 0 | 0 | 0 | 0 | MSS | 2.38 |
| P8-RLL | 3 | 1 | 0 | 2 | 0 | 0 | 0 | 0 | 0 | 0 | MSS | 3.97 |
| P8-RUL | 5 | 1 | 1 | 0 | 0 | 1 | 0 | 1 | 0 | 0 | MSS | 6.35 |
| P9-LLL | 4 | 0 | 0 | 0 | 0 | 1 | 0 | 0 | 0 | 3 | MSS | 2.38 |
| P9-RLL | 1 | 1 | 0 | 2 | 0 | 1 | 1 | 0 | 0 | 0 | MSS | 3.97 |
| P10-RLL-D | 0 | 0 | 1 | 1 | 1 | 0 | 0 | 0 | 0 | 0 | MSS | 2.38 |
| P10-RML | 2 | 2 | 0 | 0 | 0 | 1 | 1 | 0 | 0 | 1 | MSS | 3.97 |
| P10-RUL | 0 | 0 | 0 | 0 | 0 | 1 | 1 | 0 | 0 | 0 | MSS | 0.79 |
| P10-RUL-A | 2 | 0 | 1 | 0 | 0 | 0 | 0 | 0 | 0 | 0 | MSS | 1.59 |
| P11-LUL | 3 | 2 | 0 | 0 | 0 | 1 | 0 | 0 | 0 | 0 | MSS | 4.76 |
| P11-RML | 1 | 0 | 0 | 0 | 0 | 1 | 0 | 0 | 0 | 1 | MSS | 0.79 |
| P12-LUL | 1 | 0 | 1 | 0 | 0 | 0 | 0 | 0 | 0 | 0 | MSS | 0.79 |
| P12-RUL | 3 | 1 | 0 | 0 | 0 | 1 | 0 | 0 | 0 | 0 | MSS | 2.38 |
| P13-RLL | 0 | 0 | 1 | 0 | 0 | 0 | 1 | 0 | 0 | 0 | MSS | 0.79 |
| P13-RLL-1 | 0 | 0 | 0 | 0 | 0 | 0 | 0 | 0 | 0 | 0 | MSS | 0.00 |
| P13-RML | 2 | 0 | 0 | 1 | 0 | 1 | 0 | 0 | 0 | 0 | MSS | 2.38 |
| P14-RLL | 2 | 0 | 0 | 0 | 0 | 0 | 1 | 0 | 0 | 0 | MSS | 1.59 |
| P14-RUL | 2 | 0 | 1 | 0 | 0 | 0 | 0 | 0 | 0 | 0 | MSS | 1.59 |
| P15-RML | 2 | 1 | 0 | 0 | 0 | 0 | 0 | 0 | 0 | 0 | MSS | 1.59 |
| P15-RUL | 1 | 1 | 0 | 0 | 0 | 1 | 0 | 0 | 0 | 0 | MSS | 1.59 |
| P16-LUL | 0 | 0 | 0 | 0 | 0 | 0 | 1 | 0 | 0 | 0 | MSS | 0.00 |
| P16-RML | 2 | 0 | 0 | 0 | 0 | 0 | 0 | 0 | 0 | 0 | MSS | 0.79 |
| P16-RUL | 2 | 0 | 1 | 0 | 0 | 0 | 0 | 0 | 0 | 0 | MSS | 1.59 |
